# Nitrogen Efficiency in Agriculture in Europe and India

**DOI:** 10.1100/tsw.2001.385

**Published:** 2001-11-27

**Authors:** Klaas W. Van der Hoek

**Affiliations:** National Institute for Public Health and Entvironment, RIVM, Bilthoven, The Netherlands

**Keywords:** animal production, animal manure, nitrogen fertiliser, crop production, nitrogen efficiency, nitrogen surplus, human diet, EU15, India

## Abstract

Nitrogen balance sheets are useful tools for studying the quantitative aspects of nutrients. Nitrogen balance sheets have been prepared for the animal production system, crop production system, and for the agricultural sector as a whole for all 15 member states of the European Union (EU15) and for the Indian subcontinent. The EU15 and India were chosen for this study on nitrogen efficiency using balance sheets because they each occupy roughly 300 million ha of land and use about 65 kg nitrogen fertiliser per hectare of agricultural land. Balance sheets were constructed for three systems: animal production, crop production, and the agricultural sector as a whole. In addition to detailed descriptions of the nitrogen balance sheets, brief recommendations for reducing nitrogen surpluses are also given. Surprisingly, the balance sheets for crop production and the agricultural sector as a whole showed a surplus of about 60 kg of nitrogen per hectare of agricultural land.

## INTRODUCTION

Nitrogen is an essential nutrient for agricultural production. In crop production, plants require nitrogen for biomass production, while animals require nitrogen for maintenance, growth, reproduction, production of milk, eggs, wool, and labour (draft animals). Harvested biomass from crop production is used as human food or animal feed. Some types of agricultural biomass like grass and fodder, and green crops, are suitable only for ruminating animals. Some types of harvested biomass such as oil crops must be processed before human consumption, with the residues being fed to animals. Finally, some crops, like cereals, can be used for both human and animal consumption. This paper focuses on the nitrogen efficiency in three distinct systems: animal production, crop production, and the agricultural sector as a whole. Nitrogen efficiency is calculated by constructing nitrogen balance sheets for these three systems. Each balance sheet is characterised by system boundaries. The systems are interrelated; output from one balance sheet is input for another. For example, animal manure as output serves as input for fertilisation of crops.

The surplus of nitrogen on the balance sheets constructed for crop production and the agricultural sector remains behind in both systems and is subject to loss or accumulation in the soil. Nitrogen losses are in volatile form, for example, ammonia ($NH_3$), nitrous oxide ($N_2O$) and di-nitrogen ($N_2$), or in the form of nitrate ($NO_3$) leaching and runoff. Improving nitrogen efficiency in the agricultural sector lowers the nitrogen surplus on the balance sheet and also lowers nitrogen emissions.

Nitrogen balance sheets can be used on different scales, ranging from farm and catchment level to county and country level[1,2,3,4]. In this paper calculations are made for the 15 European Union states, consisting of Austria, Belgium, Denmark, Finland, France, Germany, Greece, Ireland, Italy, Luxembourg, the Netherlands, Portugal, Spain, Sweden, and the U.K., and for India. For reasons of compatibility all data used have been taken from the FAOSTAT database, with 1998 as the reference year[5].

## AGRICULTURE AND HUMAN FOOD IN THE EU15 AND INDIA

The EU15 and India each occupy roughly 300 million ha of land. Their respective use of nitrogen fertiliser is about the same when expressed per hectare of agricultural land (see Table 1). Nitrogen fertiliser is used on 30 million ha of permanent pasture in the EU15 and the average fertiliser input is 93 kg/ha of fertilised grassland[6].

There is a great discrepancy in number of inhabitants between the EU15 and India, and also in the food intake and composition of the average human diet (see Table 1). Whereas the average food intake in EU15 is 3439 cal per capita per day and the protein intake is 6.19 kg N/year, the respective corresponding values for India are 2399 cal per capita per day and 3.33 kg N/year. Moreover, the share of protein from animal products is 60% in the EU15 and 17% in India. Globally, animal products contribute 37% to the human protein consumption.

## NITROGEN BALANCE SHEET ON ANIMAL PRODUCTION

The system boundaries on the animal production balance sheet are the animals themselves, without the corresponding land area. The size of the animal population in the EU15, India, and the world is given in Table 2. The animal production balance sheet (Table 3) starts with the calculation of the amount of nitrogen contained in animal products (meat, milk, eggs, and wool). Table 4 details their production by category of animal, such as dairy cattle, chickens, and so on. The next step is the calculation of the nitrogen contained in animal manure and urine (Table 5). The nitrogen excretion rates for cattle were calculated using standard feeding tables for the minimum protein requirement for maintenance and production of milk and meat. The calculated intake of nitrogen by animals was reduced by the amount of nitrogen in animal products, under the assumption that the remaining nitrogen was excreted by the animal[7,8]. These values compare well with the literature[9,10,11,12]. The nitrogen excretion rates for pigs and poultry were taken from the Dutch literature[7,8] and are in line with other European values[13,14,15,16]. With regard to biological production, it is assumed that pigs and poultry in India have similar excretion rates to pigs and poultry in the

**TABLE 1**
**Land Use, Fertiliser Consumption, Human Population, and Human Food Intake in EU15, India, and the World in 1998**

| | EU15 | India | World |
|---|---|---|---|
| **Area**[a] | | | |
| Total land area | 313.2 | 297.3 | 13,048 |
| Agricultural land | 142.5 | 180.6 | 4,938 |
| Arable land | 75.6 | 161.5 | 1,380 |
| Permanent crops | 10.9 | 8.0 | 132 |
| Permanent pasture | 56.0 | 11.1 | 3,427 |
| **Fertiliser** | | | |
| Total N input[b] | 9,697 | 11,354 | 82,421 |
| Total N input per agricultural land[c] | 68 | 63 | |
| **Human population**[d] | 375 | 982 | 5,901 |
| **Human food intake** | | | |
| Food intake[e] | 3,439 | 2,399 | 2,798 |
| Protein intake[f] | 6.19 | 3.33 | 4.38 |
| Vegetable products | 2.45 | 2.75 | 2.74 |
| Animal products | 3.74 | 0.58 | 1.64 |
| Share animal products | 60% | 17% | 37% |

[a] Million ha.
[b] Million kg.
[c] kg N/ha.
[d] In millions.
[e] Calories per capita per day.
[f] kg N per capita per year.

Source: FAO (2001) FAOSTAT database collections. FAO, Rome. Available on the Internet at http://www.apps.fao.org.

**TABLE 2**
**Number of Animals in EU15, India, and the World in 1998**

| Animal Category | EU15 | India | World |
|---|---|---|---|
| Cattle | 83.2 | 212.1 | 1338.8 |
| Dairy | 21.5 | 35.0 | 224.1 |
| Non-dairy | 61.7 | 177.1 | 1114.7 |
| Buffalo | 0.16 | 90.1 | 160.7 |
| Sheep | 115.9 | 57.1 | 1055.2 |
| Goats | 12.1 | 121.4 | 698.0 |
| Pigs | 120.6 | 16.0 | 880.4 |
| Chickens | 1,005 | 375 | 13,219 |
| Horses | 2.2 | 1.0 | 58.5 |
| Asses | 0.41 | 1.0 | 43.2 |
| Mules | 0.22 | 0.20 | 13.5 |
| Camels | — | 1.0 | 19.2 |

*Note:* Numbers are given in millions.

Source: FAO (2001) FAOSTAT database collections. FAO, Rome. Available on the Internet at http://www.apps.fao.org.

**TABLE 3**
**Nitrogen Balance Sheets for Animal Production, Crop Production, and the Agricultural Sector in EU15 and India in 1998**

| EU15 INPUT | | EU15 OUTPUT | | INDIA INPUT | | INDIA OUTPUT | |
|---|---|---|---|---|---|---|---|
| **Animal Production** | | | | **Animal Production** | | | |
| Feedstuffs | 4.227 | Animal products | 2.167 | Feedstuffs | 3.266 | Animal products | 0.625 |
| Consumed grass | 6.378 | Animal manure | 8.439 | Consumed grass | 1.337 | Animal manure | 15.404 |
| | | | | Consumed in forests | 4.000 | | |
| Unaccounted-for N | | | | Unaccounted-for N | 7.426 | | |
| Total | 10.606 | Total | 10.606 | Total | 16.029 | Total | 16.029 |
| **Crop Production** | | | | **Crop Production** | | | |
| Fertiliser | 9.697 | Harvested crops | 4.705 | Fertiliser | 11.354 | Harvested crops | 6.039 |
| Animal manure | 8.439 | Consumed grass | 6.378 | Animal manure | 5.135 | Consumed grass | 1.337 |
| N-fixation | 1.300 | | | N-fixation | 1.650 | | |
| Unaccounted-for N | | Surplus | 8.353 | Unaccounted-for N | | Surplus | 10.763 |
| Total | 19.436 | Total | 19.436 | Total | 18.139 | Total | 18.139 |
| **Agricultural Sector** | | | | **Agricultural Sector** | | | |
| Fertiliser | 9.697 | Animal products | 2.167 | Fertiliser | 11.354 | Animal products | 0.625 |
| N-fixation | 1.300 | Crop products | 2.384 | N-fixation | 1.650 | Crop products | 5.611 |
| Feedstuffs | 1.906 | | | Feedstuffs | 6.838 | Animal manure | 10.269 |
| Unaccounted-for N | | Surplus | 8.353 | Unaccounted-for N | 7.426 | Surplus | 10.763 |
| Total | 12.904 | Total | 12.904 | Total | 27.268 | Total | 27.268 |

*Note:* Amounts given in Tg N (1 Tg = $10^{12}$ g).

**TABLE 4**
**Animal Production in EU15, India, and the World in 1998**

| Animal Category | EU15 | India | World |
|---|---|---|---|
| **Meat Production** | | | |
| Beef and veal | 7647 | 1400 | 55,078 |
| Buffalo | 1.5 | 1380 | 2949 |
| Horses | 87 | | 660 |
| Sheep | 1065 | 226 | 7403 |
| Goats | 89 | 462 | 3479 |
| Pigs | 17,711 | 543 | 87,647 |
| Chickens | 8757 | 540 | 61,688 |
| **Milk Production** | | | |
| Dairy cattle | 121,965 | 35,500 | 483,060 |
| Buffalo | 156 | 35,850 | 58,200 |
| Sheep | 2186 | | 8067 |
| Goats | 1560 | 3150 | 12,032 |
| **Egg Production** | 5368 | 1658 | 48,000 |
| **Wool Production** | 182 | 44 | 2371 |

*Note:* Amounts given in million kg.

Source: FAO (2001) FAOSTAT database collections. FAO, Rome. Available on the Internet at http://www.apps.fao.org.

**TABLE 5**
**Nitrogen Excretion (in kg N) Per Animal Per Year for the Main Animal Categories**

| Animal Category | EU15 | India |
|---|---|---|
| Dairy cattle | 100 | 60 |
| Non-dairy cattle | 50 | 40 |
| Young cattle | 46 | 25 |
| Suckling cows | 80 | 60 |
| Beef cattle | 40 | |
| Draft cattle | | 40 |
| Buffalo | | 45 |
| Sheep | 10 | 10 |
| Goats | 9 | 9 |
| Pigs | 11 | 11 |
| Chickens | 0.5 | 0.5 |
| Horses | 50 | 45 |
| Camels | | 55 |

Sources: Van der Hoek, K.W. and Couling, S. (1996) Manure Management, SNAP code 100500. In Joint EMEP/CORINAIR Atmospheric Emission Inventory Guidebook. 1st edition. G. McInnes, Ed. European Environment Agency, Copenhagen. p. B1050.1–B1050.16. and Van der Hoek, K.W. (1998) Nitrogen efficiency in global animal production. *Environ. Pollut.* **102** Suppl. 1, 127–132.

EU15. In summing, the amount of nitrogen in animal products and animal manure in this study was assumed equal to the total nitrogen intake in feed ingested by all animals.

Partitioning feed nitrogen between feedstuffs and consumed grass is based on assumptions on the share of consumed grass in the total intake. For the EU15, cattle are assumed to have a share of 80%, whereas for horses, sheep, and goats this share is 95%. The remaining nitrogen for these animal categories is produced as feedstuffs. The feed demand of pigs and chickens is supplied by 100% feedstuffs. The calculated nitrogen in consumed grass corresponds with an animal intake of 115 kg N/ha of grassland, which is a reasonable figure. The animal feeding situation in India is based on a literature survey[17,18,19,20,21,22,23]. The share of grass that the animals consume is assumed at 10% for cattle, horses, sheep, and goats, and 5% for buffalo. The share of feedstuffs in the ration is assumed to be 5% for sheep and goats, 10% for cattle and buffalo, and 67% for pigs and chickens. The feedstuffs are supplemented with crop residues, assumed to be 25% of the harvested crops. Animals grazing in forests consume grass with an estimated nitrogen uptake of 4 Tg, about three times more than the uptake from permanent pastures. Totalling feedstuffs and consumed grass in pastures and forests shows that 7426 Tg N is not accounted for. Home gardens, backyard plots, and residues from the kitchen, roadsides, and arable land provide part of this unaccounted-for nitrogen. In the balance sheet the amount of unaccounted-for nitrogen is 46% of the total calculated animal intake. This value is comparable with other studies, which show protein deficits of up to 50%[21,24].

The nitrogen efficiency is calculated as the ratio of nitrogen output in products to nitrogen input in feed. From the animal production sheet it can be calculated that nitrogen efficiency values for EU15 and India are 20 and 4%, respectively. The low value for India is explained by the relatively low beef and veal meat production and by the use of buffalo as draft animals. Draft animals produce power and consume feed but do not gain weight[25].

## NITROGEN BALANCE SHEET ON CROP PRODUCTION

The second balance sheet shown in Table 3 comprises crop production, which includes harvested crops and consumed grass. It is emphasised that only the amounts of nitrogen that effectively leave the arable field or pasture are taken into account. This means that crop residues left in the field and spilt grass are not considered. Crop production in the EU15, India, and the world is presented in Table 6, while the amount of consumed grass has already been discussed in the foregoing section. The nitrogen supply in the EU15 consists of fertiliser, animal manure, and N fixation, this last factor based on a global N fixation of 45 Tg of nitrogen[26]. The amount of fixed nitrogen in the EU15 and India is assumed to be proportional to the agricultural land. For India it is assumed that only 33% of the manure is used as fertiliser[27,28]. Some of the manure is produced outside the crop production system, in forests and on roadsides, while another part is used as fuel and thus leaves the crop production system.

Nitrogen efficiency is indicated by the ratio of nitrogen output in harvested crops and consumed grass to nitrogen input in fertiliser, animal manure, and nitrogen fixation. From the crop production sheet (Table 3) nitrogen efficiency in the EU15 and India is calculated at 57 and 41%, respectively. The lower value for India can most likely be explained by the relatively high fertiliser losses in rice production due to high ammonia volatilisation losses caused by application of urea fertiliser[29].

## NITROGEN BALANCE SHEET ON THE AGRICULTURAL SECTOR

The third balance sheet envelops all agricultural activities (see Table 3). Most entries are taken from the animal and crop production sheets. Part of the harvested crops are fed to animals and another, smaller, part is used as seed for the next crop; these quantities do not leave the agricultural sector. The amounts for feed and seed are taken from the FAO Commodity Balances and subtracted from the harvested crops[5]. The animal production balance sheet shows the required amount of feedstuffs. The amount not delivered by harvested crops has to be supplied from outside the agricultural sector (for example, from by-products of food and oilseed processing).

The amount of feedstuffs on the balance sheet for India includes the nitrogen uptake in forest grazing. As mentioned in the previous section, only 33% of the animal manure is used as fertiliser in crop production, so 67% of the manure is recorded as an output entry on the balance sheet. Nitrogen efficiency is defined as the ratio of nitrogen output in products leaving the agricultural sector to nitrogen input from an external origin, such as fertiliser, nitrogen fixation, and external animal feed. The overall nitrogen efficiency in the agricultural sector of the EU15 is 35%; for India, 23%. With reference to the remarks already made on the animal and crop production sheets, this sounds reasonable.

## THE FATE OF THE NITROGEN SURPLUS

The nitrogen balance sheets for animal production, crop production, and the agricultural sector, as discussed in previous sections, are presented in Table 3. The balance sheets from crop production and the agricultural sector as a whole display the same surplus quantity. This is not surprising because their system boundaries are the same: total agricultural land. This phenomenon of equal surpluses can be used in reverse as a check on the calculations. Dividing the surplus by the total agricultural land results for the EU15 in a surplus of 59 kg N/ha and for India, in 60 kg N/ha. These are average figures, but it is evident that both EU15 and India have locations with higher surpluses per hectare as well as locations with lower surpluses per hectare. The nitrogen surplus is subject to loss as $NH_3$, $N_2O$, $N_2$, and $NO_3$; the remainder is accumulated in the soil. Ammonia emissions present an environmental threat, and volatilised ammonia means a financial loss for the farmer[29,30]. Ammonia emissions are not taken into account in these balance calculations. The majority of the emitted ammonia will be deposited on agricultural lands. The nitrogen in the ammonia leaving the agricultural system is assumed to be replaced by $NO_x$ deposition. EU15 and Indian greenhouse

**TABLE 6**
**Crop Production in EU15, India, and the World in 1998**

| Crop Category | N Content[a] | EU15 | India | World |
|---|---|---|---|---|
| Cereals | 16 | 213,649 | 226,946 | 2,081,766 |
| Starchy roots | 3 | 43,750 | 24,848 | 641,669 |
| Sugar crops | 2 | 115,260 | 262,090 | 1,522,824 |
| Pulses | 35 | 5827 | 12,972 | 56,032 |
| Oil crops | | | | |
| Soybeans | 59 | 1582 | 6942 | 159,957 |
| Groundnuts | 40 | 3 | 9160 | 34,320 |
| Sunflower seed | 28 | 3589 | 1170 | 25,318 |
| Rapeseed | 35 | 9556 | 4703 | 35,842 |
| Seed cotton | 30 | 1523 | 6223 | 51,723 |
| Vegetables | 2 | 53,698 | 57,096 | 624,271 |

*Note:* Amounts given in million kg.

[a] Nitrogen content in crop products is expressed as g N/kg and values are taken from the literature[52,53].

Source: FAO (2001) FAOSTAT database collections. FAO, Rome. Available on the Internet at http://www.apps.fao.org.

gas emissions of agricultural origin amount to 683 and 234 Gg $N_2O$, and to 8551 and 14,760 Gg $CH_4$, respectively[30,31,32,33]. Converted to $CO_2$-equivalents, the agricultural emissions in the EU15 and India are 391 and 382 Tg $CO_2$-equivalents, respectively.

Nitrate leaching affects the quality of the groundwater. While standards on the nitrate content are set to ensure the use as drinking water for humans and animals, high levels of nitrate are found in the EU15 as well as in India [3,34,35].

## CLOSING THE NITROGEN CIRCLE

Much research is devoted to more efficient nutrient use in agriculture. Some research focuses on improvement of animal production systems[17,36,37,38,39,40] and crop production systems[41]. Other research is devoted to the role of animals in global food production [42,43] and global food production in the next decades[44,45,46].

Promising means for improving nitrogen efficiency are (1) more efficient use of nitrogen fertiliser and animal manure by preventing loss of ammonia[29,47] and (2) more efficient use of animal feeds by providing feed to growing animals in sufficient quantities (feeding above maintenance level of the animal means that feed is available for production of meat and milk)[8,17].

Another aspect is the ratio of animal to vegetable products in the human diet[48]. Changing the human diet in European countries by lowering the share of animal protein will decrease the demand for and production of animal products, which will, in turn, lead to increased nitrogen efficiency in the agricultural system.

Finally, environmental costs of animal and crop products differ in terms of greenhouse gas emissions[49,50,51]. Beans, potatoes, and cereals have relatively low greenhouse gas emissions, followed by tomatoes grown in greenhouses; pork and rice have relatively high greenhouse gas emissions. The composition of the human diet therefore has an impact on global climate change.

| | | |
|---|---|---|
| Received: | July | 28, 2001 |
| Revised: | November | 12, 2001 |
| Accepted: | November | 12, 2001 |
| Published: | November | 27, 2001 |

## BIOSKETCH

**Klaas W. Van der Hoek** is senior scientist at RIVM, the Dutch National Institute for Public Health and the Environment in Bilthoven. His activities include the coordination of the agricultural emission data for the annually published Environmental Balance. Klaas graduated with a M.Sc. in food technology from the Wageningen Agricultural University, The Netherlands in 1975. After working for 15 years on environmental issues in several national extension services of the Dutch Ministry of Agriculture, Nature Management and Fisheries he assumed a position in 1991 at RIVM. His activities there have included Conference Secretary and Proceedings Editor for the First International Nitrogen Conference held in Noordwijkerhout, The Netherlands in March 1998. His current research interests are nutrient balances, sustainable animal and crop production systems, and use of animal manure.
